# Electromechanical behaviors of a PVDF beam-film coupled energy harvester under droplet impact

**DOI:** 10.1371/journal.pone.0319751

**Published:** 2025-04-04

**Authors:** Weijia Yang, Guannan Hao, Zhinan Li, Shuai Zhang, Lixin Lu

**Affiliations:** College of Mechanical and Electrical Engineering, Qingdao University, Qingdao, China; inSync Mirror LLC, UNITED STATES OF AMERICA

## Abstract

Droplet-based harvesters can effectively transfer the kinetic energy of impacting water droplet into electrical energy. Based on conventional cantilever structure of harvesters, a beam-film coupled structure is developed by extending the piezoelectric film along the beam length to enhance the power generation. First, the droplet impact force is modeled based on impulsive theorem, which is applied in constructing the simulation model of the proposed harvester using COMSOL. Second, the developed model is validated by comparing with experimental results. Then the dynamic response and electromechanical coupling effect of the proposed structure under droplet impact are comprehensively analyzed. Results show that the energy density of beam-film coupled structure with film ratios 0.6 and 0.8 is much higher than that of cantilever structure, showing an increase of 522.67% and 645.18% respectively. Moreover, the harvester with film ratio 0.6 always generates greater total electrical energy collected than the film ratio 0.8, and the difference between them becomes more significant for higher impact velocities> 1.25m/s and larger droplet diameters > 3.2mm. Results also show that there exist optimal structural parameters of the harvester which lays a foundation for further structural optimization to continuously improve the energy conversion efficiency.

## 1. Introduction

Scavenging energy from the environment has become a key concern in addressing issues such as the growing global demand for renewable energy and the environmental pollution caused by traditional fossil fuel consumption. Based on this issue, Ahmad [1] explored global energy demand trends and analyzed future changes in energy supply and consumption. Furthermore, Muhammad et al. [2] analyzed the impact of renewable and non-renewable energy consumption on global economic growth and advocated promoting renewable energy for sustainable development. In addition to traditional environmental energy sources such as wind [3], solar [4], and tidal energy [5], the harvesting of environmental vibration energy using piezoelectric materials has attracted considerable attention in recent years. Such vibrations, generated by fluid flow, mechanical system oscillations, and other similar phenomena, can be effectively harvested using vibration energy harvesting systems. This method has been the subject of extensive research by numerous scholars. Zhang et al. [6] proposed a piecewise quad-stable nonlinear piezoelectric energy harvester, which introduces flexible linear stiffness baffles. This enhancement boosts the energy harvesting capabilities of the harvester in collecting ultra-low frequency energy. Huang et al. [7] proposed a novel bionic-dipteran magnetoelastic bi-stable dynamic vibration absorber, which achieves broadband TET and energy conversion performance under weak ambient vibrations. Huang [8] proposed a nonlinear energy conversion and multi-stiffness combination inspired dynamic vibration absorber that demonstrates superior targeted energy transfer and energy conversion performance. Huang et al. [9] proposed an electromagnetic bi-stable energy harvester (EBEH) with tunable stiffness for scavenging hydrokinetic energy from flow-induced vibration (FIV) scenarios. This structure exhibits higher energy conversion efficiency and targeted energy transfer capacity within a certain range of inlet velocities. Most of the studies focus on harvesting energy from intense environmental vibrations, while the collection of energy from weak vibrations, particularly subtle impacts, remains an area that requires further exploration.

As a clean energy source, rainfall contains a substantial amount of energy, mainly consisting of the kinetic energy and electrical potential energy of falling raindrops. Therefore, droplet-based harvesters are being widely investigated for rain energy harvesting. This is especially true in scenarios where the use of solar-based power generation devices is often constrained. Ahmad et al. [10] confirmed that the energy contained in rainwater can meet the power supply requirements of low-energy consumption devices, such as remote and wireless sensors. Paradiso et al. [11] summarized various forms of vibration energy harvesting and demonstrated the feasibility of renewable energy as a replacement for traditional chemical batteries. This approach also helps avoid issues such as short lifespan, high replacement and maintenance costs, and environmental pollution. Aiming at collecting kinetic energy of falling raindrops, Hao et al. [12] focused on the droplet impact process, analyzing the effect of droplet dynamics on the output of PVDF (Polyvinylidene Fluoride) cantilevers.

As the impact force induced by water droplet is usually small, rendering traditional ceramic piezoelectric materials like PZT (Lead Zirconate Titanate) unsuitable for harvesting the weak energy in droplets. By comparison, highly flexible polymer materials like PVDF show significant potential in droplet-based harvester applications due to its lightweight, high flexibility, and impedance. Erturk et al. [13] explained that PVDF’s polarized β-phase structure gives it a piezoelectric coefficient (d33) of 20–30 pC/N. This makes PVDF highly efficient for low-frequency vibration energy harvesting, surpassing rigid ceramics like PZT. Ma et al. [14] and Sukumaran et al. [15] explored the feasibility of using PVDF piezoelectric material for energy harvesting from water droplets. The research conducted by Tansel [16] and Rakhmankulov et al. [17] thoroughly demonstrated the thermal stability, superior malleability, impact resilience, and flexibility of PVDF materials. Kerbow [18] also demonstrated the reliability and durability of PVDF materials. Particularly, Walter et al. [19] described PVDF’s excellent mechanical flexibility, allowing it to withstand significant bending and stretching without cracking and making it ideal for wearable and flexible devices. By contrast, PZT and similar ceramic materials often fail under such stresses. Zhang et al. [20] also mentioned that PVDF’s chemical stability allows it to function reliably in harsh conditions, such as acidic or high-humidity environments, extending device lifespans and reducing waste. This shows great potential of PVDF-based polymer materials in outdoor utilizations such as rain energy harvesting scenarios.

As the use of piezoelectric polymers is experiencing explosive growth in these decades, the impact of materials on the environment and concerns on the recycling after reaching their lifespan have also attracted widespread attention. For the toxicological aspects of PVDF, Erturk et al. [13] emphasize that PVDF’s lead-free nature eliminates the environmental and health risks associated with PZT, making it safe and eco-friendly. Vallem V et al. [21] also praise PVDF’s chemical stability and non-toxic nature, which make it an eco-friendly option for medical and environmentally sensitive applications. In addition, Kong et al. [22] indicate that PVDF’s low processing temperature (110–135°C) can significantly reduce carbon emissions, contrasting with PZT’s sintering process (>1200°C). For treatments when materials reach their lifetime expectance, two primary recycling methods are currently employed: mechanical recycling and chemical recycling. In terms of mechanical recycling, there exist professional companies which provide essential services like shredding, granulating, and pelletizing PVDF materials. These processes allow PVDF to be re-utilized in various applications without altering its chemical properties, ensuring that its performance remains intact even after recycling. For chemical recycling, there are notable advancements as well. For instance, Arthur et al. [23] explore chemical processes for breaking down PVDF into monomers that can be repolymerized. This approach provides new possibilities for high-purity material recovery.

In the last decade, PVDF-based harvesters have been extensively investigated and great efforts have been made in developing novel structures to improve the energy conversion efficiency and to expand their flexibility of utilizations [24–26]. However, toward rain energy harvesting utilizations, the real rainfall has randomness and uncertainty. If the energy harvester is implemented directly under actual rainfall condition, it will be excited by successive impacts of raindrops with random droplet size or impact position, making the harvester less efficient. So the concept of droplet-based harvester design is transiting from “direct collection” to “first collection, then utilization” which is especially advantageous for regions with almost no or very little rainwater. As a promising approach, more and more devices are designed with a container for collecting rainwater followed by a control unit to generate droplet with specific size and velocity. For instance, Doria et al. [27] proposed a harvester with a cantilever beam equipped with a tip spoon filled with water. Bao and Wang [28] developed a rooftop rainwater energy harvester equipped with a passive rainwater buffer installed above a piezoelectric cantilever beam which was tested under different flow rates. Various designs of energy harvester structures have been proposed by far, such as cantilever [29], bridge [30], and floating circle [31], the cantilever structure remains the most efficient and compact, demonstrating superior electrical output. In particular, when a droplet impacts the beam tip, a large strain is generated, even with moderate impact force, which is directly proportional to the voltage output in an open-circuit condition [24]. In this work, we intend to provide an improved structure based on cantilever beam by extending the PVDF film along the beam length. Unlike traditional designs with localized or segmented PVDF films, the proposed structure utilizes the beam-film coupling effect. This coupling optimizes the mechanical deformation of the PVDF film, improving the efficiency of electromechanical energy conversion. The focus of this study is the extended PVDF film design, which enables a larger beam surface to participate in energy harvesting. This design is particularly effective for low-frequency and distributed raindrop impacts. The extended film configuration increases energy collection efficiency and ensures more stable and reliable energy output under repetitive, low-frequency impacts. Similar structure has already been investigated for the wind energy harvesters by using PZT material [32,33]. However, the focus was on the flutter motion of PZT cantilever at a low wind speed, and the brittleness of PZT can cause fatigue failure under high frequency cyclic load especially in outdoor applications. In addition, the wind-induced vibration harvesters are also completely different with that of droplet.

Many prototypes of droplet-based harvesters previously developed are investigated by means of experiments [34,35], which is related to the complexity of providing an accurate droplet-induced force model. The first model of droplet impact on PVDF sheet developed by Guigon was simplified as a perfect inelastic collision [36]. Subsequently, it was suggested that new surface material such as super-hydrophobic surfaces of beam structure may maximize the inelastic collision so as to improve the efficiency [37]. However, the droplet dynamics on treated beam surface as well as its effect on dynamic response of harvesters was not sufficiently discussed in previous studies. It is also unanimously agreed that no water layer is deposited on a super-hydrophobic surface after impact so that multiple impacts of droplet can be considered as the repeatability of identical single droplet impact. Thus it is essential to establish the quantitative relationship between impact parameters of droplet and force applied on harvesters for a single droplet. This will reveal the fundamental interplay between the droplet and structure. In our previous work [38], a systematical analysis concerning simple PVDF cantilever beam under excitation of water droplet was conducted by using an electromechanical model. In the model, the reaction force of droplet impacting on a super-hydrophobic surface was integrated into the equation of mechanical motion. This force was approximated as an average force applied during the typical impact duration [39]. However, in the developed force model, only the effect of impact parameters (e.g., impact velocity and droplet diameter) was considered. Actually, the beam stiffness has a significant effect on droplet-structure interaction, especially in improved structures beyond simple cantilever beams. Therefore, in this study, the impact force of droplet is comprehensively modeled by considering the coupling effect of impact and structural parameters. In this way, the time variation characteristics of impact force can be accurately represented. This force model is validated by experiments over a wide range of working conditions, which can be used in predicting the electromechanical coupling behaviors of the harvester.

## 2. Droplet-based harvester

**Fig 1:**
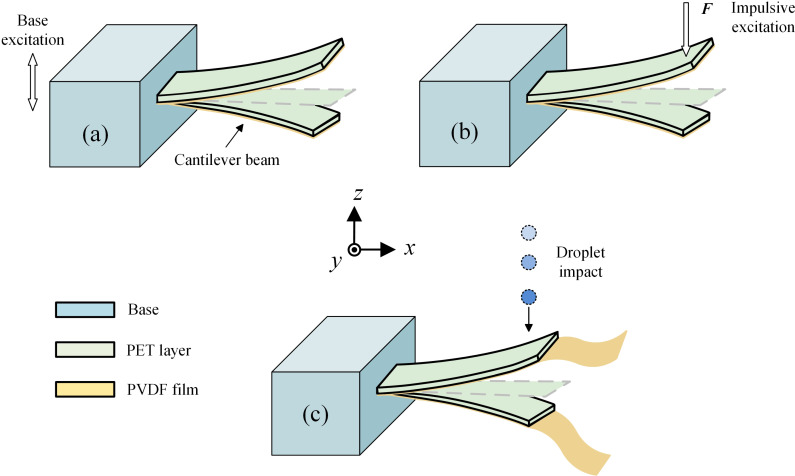
Development of beam-film coupled structure based on traditional vibration energy harvester: (a) Base excitation; (b) Droplet impact excitation; (c) Successive droplet impacts excitation.

Based on the traditional piezoelectric energy harvester (PEH) shown in Fig 1a, the widely used cantilever-style harvester is excited by base vibration. For droplet-based harvesters, the harvester is usually excited by an impulsive force *F* due to droplet impact, as shown in Fig 1b. After impact, the cantilever beam undergoes free vibration primarily dominated by the natural frequency of the beam. Further, considering the actual rainfall conditions where successive and multiple impacts of raindrops usually occur, the PVDF film is extended from the cantilever end, generating coupled structure with variable stiffness, as shown in Fig 1c. Under successive impacts of droplet, the coupling effect of flexible beam and relatively soft PVDF film leads to a higher performance on electrical outputs compared to single cantilever in Fig 1b, and the harvester vibration is enhanced especially when the impact frequency is consistent with the equivalent frequency of the structure.

**Fig 2:**
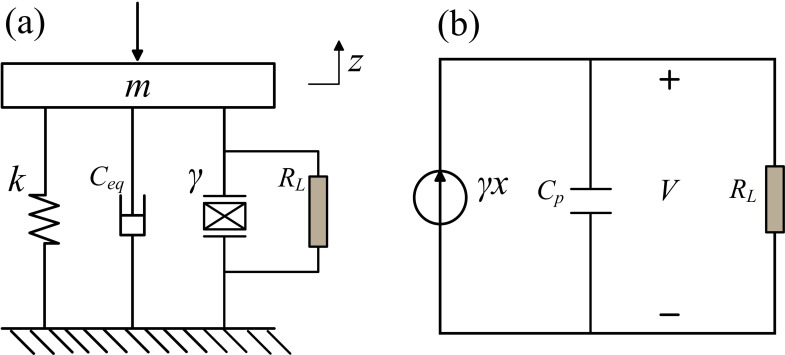
(a) Electromechanical coupling model; (b) External harvesting circuit.

The basic cantilever-style harvester is mainly composed of a PVDF film layer and a PET (Polyethylene Terephthalate) substrate layer, while the PVDF layer greatly affects the energy density collected and the PET layer primarily determines the structural stiffness. Using the equivalent lumped parameter model to describe the electromechanical coupling behavior of the coupled structure, the external harvesting circuit is shown in Fig 2.

In Fig 2, *m* and $C_{\text{eq}}$ are the equivalent mass and the equivalent damping, respectively; *k* is the stiffness of the cantilever beam; γ is the electromechanical coupling coefficient; *F* is the impact force of water droplet; *V* is the voltage output; $R_{\text{L}}$ is the external load resistance; $C_{\text{p}}$ is the internal capacitance of the cantilever beam.

Based on Newton’s second law and Kirchhoff’s law, the electromechanical governing equations of the PVDF cantilever beam can be derived as follows:


$$\{\begin{array}{c} m\overset{"}{z}+c_{eq}\dot{z}+kz+\gamma V=F\\ C_{p}\dot{V}+\frac{V}{R_{L}}=\gamma \dot{z} \end{array}$$ (1)

When subjected to impact force of droplet, the response of piezoelectric beam is primarily characterized by the d31 mode, which is more suitable for lower-intensity external stresses. The constitutive equations of piezoelectric beam can be written as:


$$\begin{array}{l} S_{ij}=s_{ijkl}^{E}T_{kl}+d_{kij}E_{k}\\ D_{i}=d_{ikl}T_{kl}+\varepsilon_{ik}^{T}E_{k} \end{array}$$ (2)

where $S_{ij}$ represents stress, $T_{kl}$ represents strain, $E_{k}$ is the electric field, and $D_{i}$ represents electric displacement. The superscripts *E* and *T* indicate the elastic compliance at constant electric field and the permittivity at constant stress, respectively. The cantilever can be approximated as Euler-Bernoulli beam so that the axial strain is proportional to the beam curvature which can be calculated through the transversal displacement of the beam:


$$S_{1}(x,z,t)=-x\frac{\partial^{2}z(x,t)}{\partial x^{2}}$$ (3)

Based on Eqs. (1) to (3), the electromechanical coupling behaviors of the harvester can be derived and analyzed in a theoretical way once the excitation force from impacting droplet is determined.

## 3. Droplet impact force

The interaction between water droplet and beam surface upon impact process is transient and variable. Existing force model of impacting droplet is developed based on impact parameters, and the effect of structural parameters is not considered. However, the coupling effect of these parameters has a great impact on dynamic response so as the energy generation of the droplet-based harvesters. To develop a more comprehensive model for droplet impact force, the impulse theorem is applied by considering both the impact parameters of droplet and structural parameters of the beam-film coupled structure.

**Fig 3:**
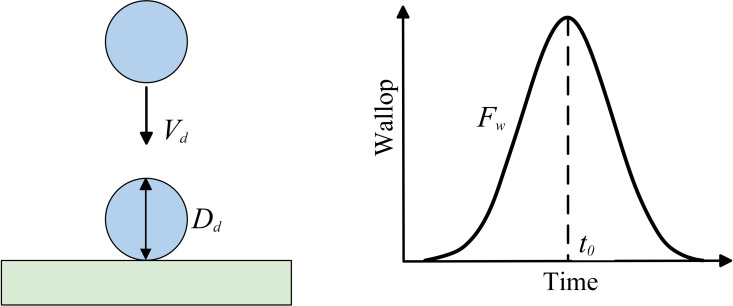
Droplet impact process and distribution curve.


As shown in Fig 3a, the short impact duration of droplet is $t_{\text{d}}$ which can be simplified as the crash of droplet that the droplet passing through a distance of $D_{\text{d}}$ with the impact velocity of $V_{\text{d}}$, written as:

$$t_{d}=\frac{D_{d}}{V_{d}}$$ (4)

According to the impulse theorem, the impact force $F_{t}$ applied by water droplet on beam surface can be approximated as a constant force calculated by:


$$F_{t}=\frac{m_{w}V_{d}}{t_{d}}$$ (5)

where $m_{\text{w}}$ is the mass of the droplet expressed as:


$$m_{w}=\frac{4}{3}\rho_{w}\pi r_{d}^{3}=\frac{1}{6}\rho_{w}\pi D_{d}^{3}$$ (6)

with $\rho_{\text{w}}$ the density of water.

To accurately characterize the droplet dynamics with first increasing and then decreasing during impact process, a Gaussian function is employed to represent the variation of the impact force over time during the impact process shown as below:


$$f(t)=\frac{1}{\sqrt{2\pi t_{d}}}\exp\left[-\frac{{\left(t-t_{0}\right)}^{2}}{2t_{d}^{2}}\right]$$ (7)

where $t_{0}$ is the time instant related to the peak value of force, shown as in Fig 3b, which is associated with the structural stiffness expressed as:


$$t_{0}=1/(10.92E^{*})$$ (8)

with $E^{*}$ the equivalent elastic modulus of the coupled harvester, GPa. The elastic modulus of the cantilever-style harvester can be derived from the natural frequency $f_{0}$ which can be written as:


$$f_{0}=\frac{1.8751^{2}}{2\pi }sqrt(\frac{E^{*}I}{\rho Ab^{4}})$$ (9)

where *b* is the beam width; *ρ* is the density of the harvester which can be obtained by the following expression:


$$\rho ∼\frac{h_{1}}{h_{1}+h_{2}}\rho_{1}+\frac{h_{2}}{h_{1}+h_{2}}\rho_{2}$$ (10)

In Eq. (9), *A* is the equivalent cross-sectional area which can be obtained by the following expression:


$$Ay_{c}=\sum A_{\text{i}}y_{ci}$$ (11)

where $A_{i}$ is the cross-sectional area of the materials *i* (i.e., PET layer and PVDF layer); $y_{\text{c}i}$ is the neutral axis of the two different materials. *I* is the moment of inertia which can calculated by:


$$I=\sum (\frac{h_{i}b_{i}^{3}}{12}+A_{i}a_{i}^{2})$$ (12)

where $y_{\text{c}}$ is the neutral axis of the harvester; $h_{i}$ is the thickness of the material *i*; $b_{i}$ is the width of the material *i*; $a_{i}$ is the distance from the neutral axis of the material *i* to the neutral axis of the harvester. The cantilever beam used in the harvester is a composite beam made of two materials. Assuming the width of the beam substrate is $b_{1}$, the equivalent width of PVDF, $b_{2}$, can be determined using the equivalent section method as follows:


$$b_{2}=\frac{E_{2}}{E_{1}}b_{1}$$ (13)

By combining the aforementioned equations (4) to (13), the impact force of droplet can be determined as:


$$F_{w}=F_{t}\cdot f(t)$$ (14)

The time variation characteristic of the force model is shown in Fig 3b. By using this Gaussian-type force model, the impact dynamics of droplet can be effectively reflected and the involvement of multiphase flows in establishing the simulation model in the following section can also be avoided.

## 4. Simulation analysis

### 4.1. Model validation

#### 4.1.1. Simulation model.

**Fig 4:**
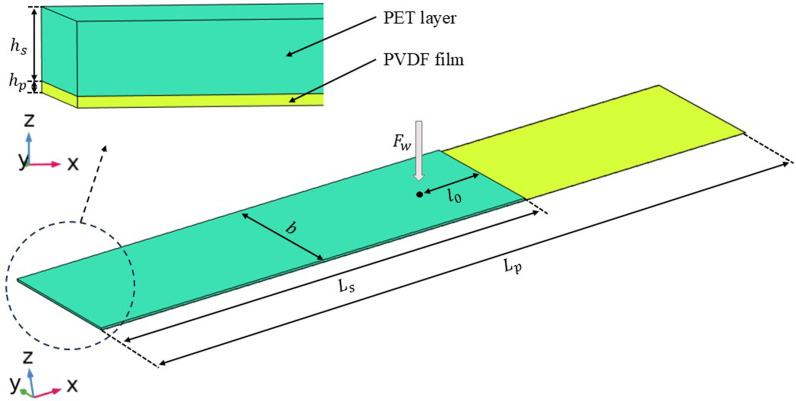
Simulation model for beam-film coupled harvester.

By applying the Gaussian force model, the simulation model of the harvester is constructed in COMSOL Multi-physics software, shown as in Fig 4. Initial parameters used in the parametric model are shown in Table 1. By performing the modal analysis and impact response analysis under various working conditions, the characteristics and the beam-film coupling effect of the harvester can be comprehensively investigated.

**Table 1:** Main parameters of the energy harvester used in the simulation model.

Parameters	Symbol	Value	Unit
Length of cantilever beam	*L*_s_	40	mm
Width of cantilever beam	*b*	16	mm
Thickness of PET layer	*h*_s_	0.18	mm
Thickness of PVDF layer	*h*_p_	0.028	mm
Elastic modulus of PET	*E*_s_	13	GPa
Elastic modulus of PVDF	*E*_p_	3.8	GPa
Bulk density of PET	*ρ*_s_	1380	Kg ∙ m^-3^
Bulk density of PVDF	*ρ*_p_	1780	Kg ∙ m^-3^
Rayleigh damping factor	α	3.5	–
Rayleigh damping factor	β	0.0001	–

The elastic matrix of the PVDF film used in the model is as follows:


$$\left[c\right]=\left[\begin{array}{cc} \begin{array}{ccc} 3.80 & 1.90 & 0.90\\ 1.90 & 3.80 & 0.90\\ 0.90 & 0.90 & 2.40\\ 0 & 0 & 0\\ 0 & 0 & 0\\ 0 & 0 & 0 \end{array} & \begin{array}{ccc} 0 & 0 & 0\\ 0 & 0 & 0\\ 0 & 0 & 0\\ 2.60 & 0 & 0\\ 0 & 9.00 & 0\\ 0 & 0 & 2.42 \end{array} \end{array}\right]\left(GPa\right)$$ (15)

The dielectric constant matrix of PVDF film is:


$$\left[\varepsilon \right]=\left[\begin{array}{ccc} 5.1 & 0 & 0\\ 0 & 8.3 & 0\\ 0 & 0 & 5.1 \end{array}\right]\times 10^{-11}\left(F/m\right)$$ (16)

The coupling matrix of the PVDF film is as follows:


$$\left[e\right]=\left[\begin{array}{cc} \begin{array}{ccc} 0 & 0 & 0\\ 0 & 0 & 0\\ 0.0180 & -0.0135 & -0.0650 \end{array} & \begin{array}{ccc} 0 & -0.0388 & 0\\ -0.0388 & 0 & 0\\ 0 & 0 & 0 \end{array} \end{array}\right]\left(C/m^{2}\right)$$ (17)

#### 4.1.2. Impact experiments.

**Fig 5:**
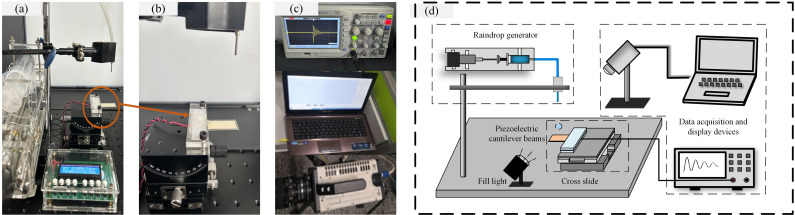
Experimental setup: (a) Droplet generation system; (b) Impact workbench for harvester; (c) Data acquisition and display system; (d) Schematic diagram of the experimental setup.

To verify the effectiveness and accuracy of the aforementioned droplet force model as well as the simulation model, impact experiments are carried out by use of artificial raindrops in laboratory conditions and experimental results obtained can be further compared with the simulation results. As shown in Fig 5, the experiment setup mainly consists of a droplet generation system, an impact workbench for harvester, and a data acquisition and display system.

The harvester is installed on the sliding support (LGX60-R) which is mounted on a 600mm ×  600mm array workbench. The commercial transducer (LDT1-028K) is used and mounted in a cantilever configuration with the same parameters set in the simulation model shown in Table 1. Additionally, the beam surface is treated with super-hydrophobic nanoparticles to ensure no water deposited on the surface, so that multiple impacts of droplet can be considered as the repeat of separated single droplet impact. The harvester is connected to an oscilloscope (SDS1000E+) to acquire the voltage output. Simultaneously, a high-speed camera (NAC HX-7s) is used to capture the dynamics of droplet and the motion of PVDF harvester, by means of which the dynamic of the whole system can be obtained and analyzed comprehensively.

**Fig 6:**
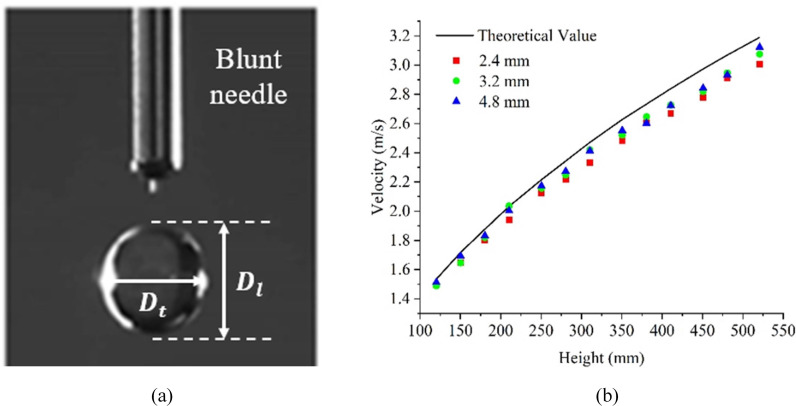
Control of impact parameters in experiments: (a) Measured droplet diameters in lateral and longitudinal directions; (b) Comparison of impact velocity between theoretical and measured values for different droplet diameters.

Water droplets are generated to simulate raindrops by using different types of blunt needles (25G, 19G and 10G) which are mounted on an iron stand. A stepper pump is used to power the syringe connected to blunt needle through a flexible tube. For multiple impact condition, the pump speed can be adjusted to adapt with the external excitation required. For accurate control of impact parameters, the droplet diameter is totally determined by the inner diameter of each blunt needle, while the impact velocity is adjusted by varying the mounting height of needle which is usually within 1.0m in laboratory. Fig 6 illustrates the droplet diameters and impact velocities measured from captured pictures by high-speed camera.

As shown in Fig 6a, in order to obtain the actual droplet size, the droplet diameter is measured at the instant when it is just squeezed from blunt needle. The empirical formula is used to determine the equivalent droplet diameter written as:


$$D_{d}=\sqrt[{3}]{D_{t}^{2}D_{l}}$$ (18)

where *D*_t_ and *D*_l_ represents the transverse diameter and the longitudinal diameter, respectively. By calculations, the droplet diameters generated by each type of blunt needle are listed in Table 2.

**Table 2:** Droplet diameters corresponding to different types of blunt needles.

Type (G)	25	19	10
Diameter (mm) (mm) factor	2.4	3.2	4.6

Fig 6b shows the measured velocities from captured pictures for different droplet diameters when the droplet releasing height is varied from 120mm to 520mm. These measured values are also compared with the theoretical values of free fall velocity, showing a high consistence between them. Additionally, the measured impact velocities of different droplet diameters (i.e., 2.4mm, 3.2mm and 4.6mm) remain basically unchanged for any releasing height of water droplet. Therefore, it can be concluded that the droplet size is totally determined by the dimension of blunt needle and has little effect on the impact velocity. The impact velocity of water droplet can be approximately obtained by the theoretical values of its free fall velocity.

**Fig 7:**
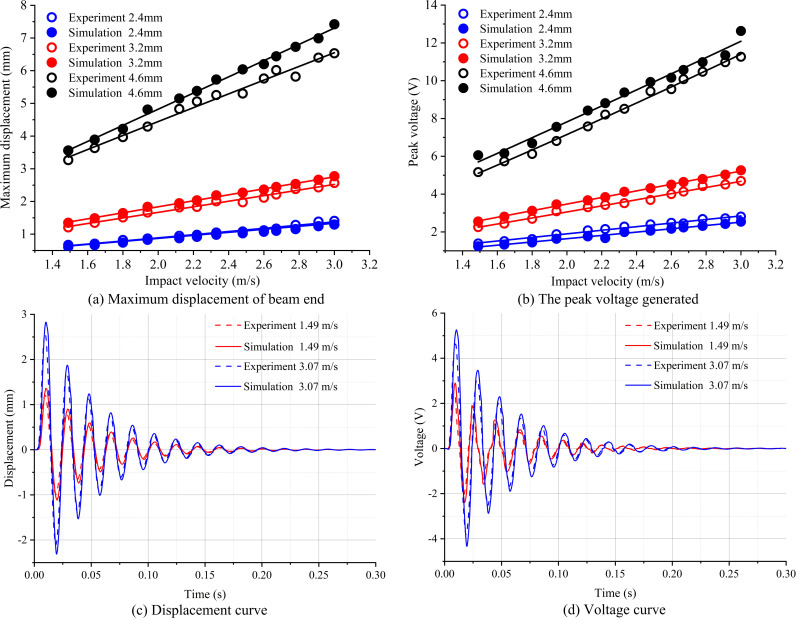
Comparison of simulation and experimental results for (a) The maximum displacement of beam end; (b) The peak voltage generated; (c) The time variation curve of beam displacement and (d) The time variation curve of voltage.

A large amount of experiments is conducted for different impact conditions. The displacement of the beam end can be obtained by the capturing of high-speed camera, while the output voltage can be measured in an open-circuit condition. These results are compared with the simulation results calculated with the same impact parameters, as shown in Fig 7.

Fig 7a and 7b compare the maximum deflection of beam end and the peak voltage generated between experimental and simulation results, respectively. For all droplet diameters, both of them show a linearity with the impact velocity. These results reveal the fact that the maximum deflection of beam end is proportional to the peak voltage in an open-circuit condition, which is also reflected by the second equation in Eq. (1) when the term of $R_{\text{L}}$ tends to zero. Besides, the simulation results are generally consistent with the experiments especially for smaller diameters 2.4mm and 3.2mm. For larger droplet of 4.6mm, results predicted by the simulation results are always slightly greater than the experiments with a maximum relative error of 13.595% for the beam deflection and 12.067% for the peak voltage respectively. It is associated with the fact of droplet splashing phenomenon which is more likely to occur for larger droplet size when the surface tension of water droplets is insufficient to counteract with the inertial force. In this case, less initial kinetic energy of droplet is transferred into strain energy of the beam structure, leading to a smaller measured value compared to the simulation results.

Fig 7c and 7d shows a comparison of the time variation curve for the beam end displacement and output voltage respectively. It can be seen that simulation results show a high consistence with the experimental results either for different droplet diameters or different impact velocities. Overall, results shown in Fig 7 confirm the accuracy and robustness of the simulation model constructed by applying the impact force model developed in section 3. Although the splash phenomenon of droplet for larger droplets is not taken into consideration in the force modeling, but the errors are still acceptable to validate the model. Besides, raindrops larger than 5.0mm are rarely seen in real rainfall conditions, thus the impact parameters selected for testing the model are also reasonable. Therefore, the simulation model coupled with the droplet impact force model can provide accurate results in predicting the electromechanical coupling behaviors of droplet-based harvesters.

### 4.2. Dynamic response

The dynamic response and vibration characteristics of the beam-film coupled harvester are greatly affected by both the impact parameters and the structural parameters. By using the simulation model, the effect of these factors is analyzed in detail.

#### 4.2.1. Effect of structural parameters.

**Fig 8:**
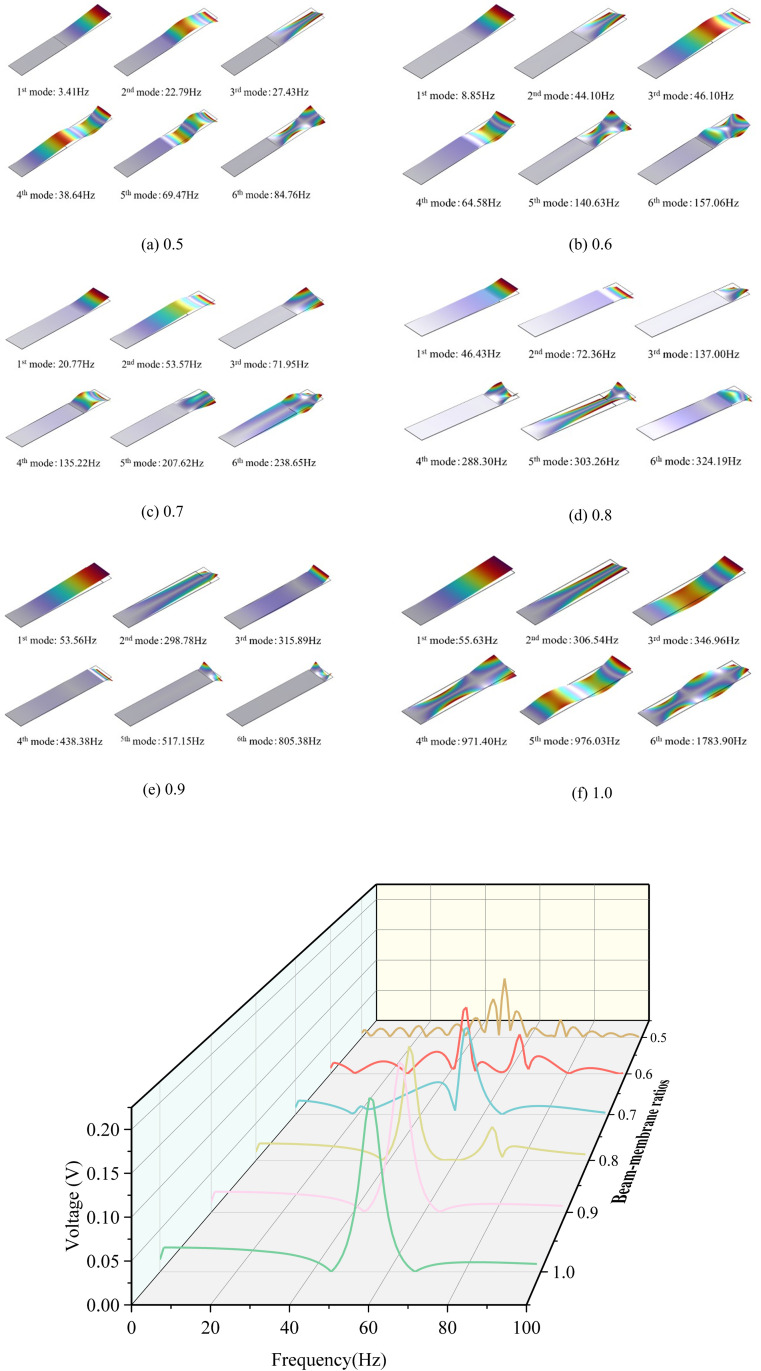
Vibration modes of the harvester with different film ratios.

The effect of structural parameters on vibration modes of the beam-film coupled harvester is analyzed by varying the PVDF film length relative to the beam length, so the film ratio *δ* is defined as the ratio of beam length on PVDF film length. For *δ* varying from 0.5 to 1.0, the first six vibration modes of the harvester calculated from the simulation model are shown in Fig 8. Noted that the film ratio of 1.0 represents the traditional cantilever structure fully covered with PVDF film.

It can be seen from Fig 8 that the natural frequency increases significantly with the film ratio. For small ratios of 0.5 and 0.6, the natural frequency is below 10Hz, representing advantages of harvesting ultra-low-frequency vibration energy. As the ratio increases, the structure shows two distinct vibration patterns: at lower-order vibrations, both the PET substrate layer and the PVDF film deform together; at higher-order vibrations, the deformation is predominantly in the PVDF film. When the ratio reaches 1.0, the harvester shows the same characteristics of vibration mode as a cantilever with a single dominant vibration mode.

**Fig 9:**
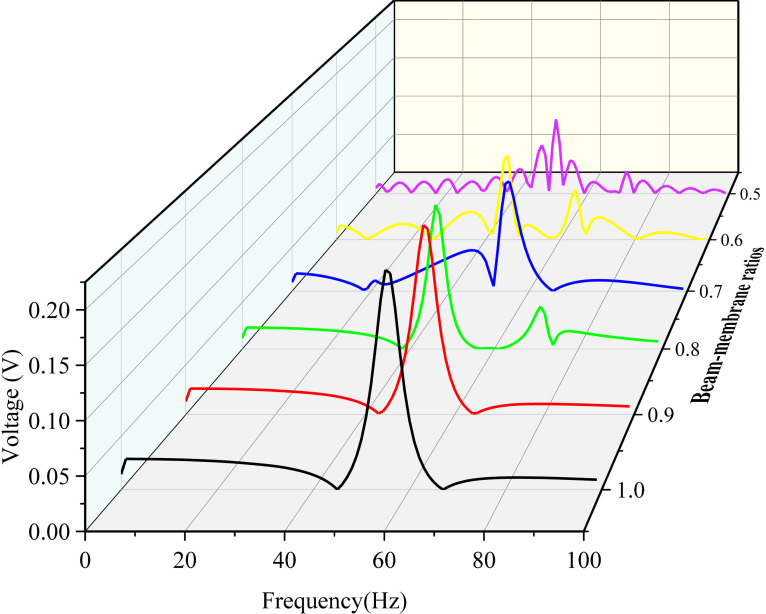
Frequency domain response curves of the output voltage under different film ratios.

By applying a droplet diameter of 3.2mm and an impact velocity of 3.0m/s for the force model, Fig 9 illustrates the voltage-frequency response curves for different *δ* values. At higher *δ* values of 0.9 and 1.0, the energy harvester primarily exhibits single-mode vibration with a peak frequency of 55.51Hz and 53.56Hz, corresponding the first mode of vibration respectively. As *δ* decreases, the vibration behavior gradually shifts to dual-mode vibration and multi-mode vibration. Although the peak voltage decreases noticeably with *δ*, the ultra-low-frequency response and multi-mode vibration characteristics still make the harvester particularly advantageous for capturing ultra-low-frequency vibration energy and varied external excitations. In particular, it shows broad bandwidth and high flexibility for specific utilizations like harvesting kinetic energy of raindrops in practical precipitation conditions.

#### 4.2.2. Energy density.

**Fig 10:**
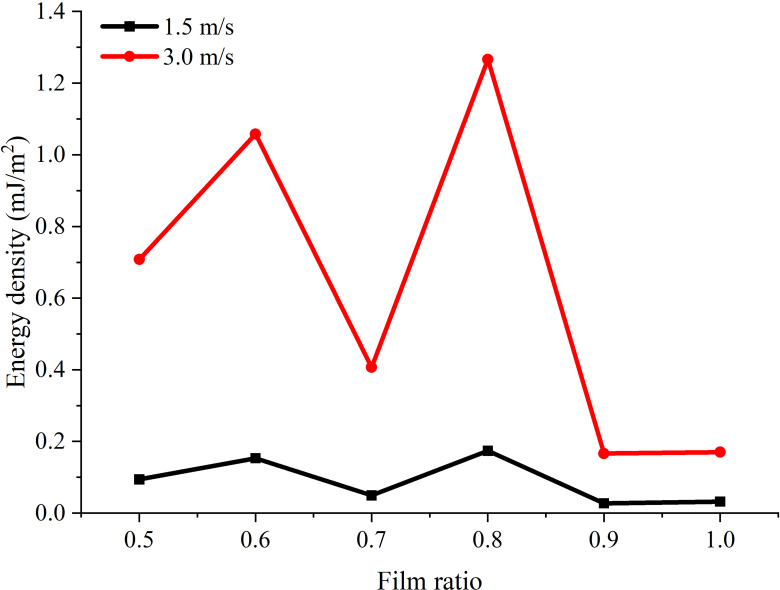
Variation of energy density with respect to film ratio under lower and higher impact velocities.

To further evaluate the performance of the beam-film coupled harvester, Fig 10 illustrates the variation of the energy density with different film ratios and impact velocities, while the energy density is defined as the total electrical energy collected per unit PVDF film area to reflect the power generation efficiency of piezoelectric materials. At lower impact velocity (e.g., 1.5m/s), there is a global decreasing trend of energy density as the film ratio increases. However, the value with film ratios of 0.6 and 0.8 are relatively higher than other film ratios. When the impact velocity is increased to 3.0m/s, the energy density of film ratios 0.6 and 0.8 is significantly increased and much higher than other film ratios, showing a peak value of 1.05786 ∙ 10^-6^mJ/m^2^ and 1.26600 ∙ 10^-6^mJ/m^2^ respectively.

Noted that the harvester with film ratio of 1.0 reproduces the traditional cantilever structure fully covering with PVDF material. According to Fig 10, the proposed harvester with film ratios of 0.6 and 0.8 presents an increase of 522.67% and 645.18% respectively compared to the cantilever structure (*δ* = 1.0) for an impact velocity of 3.0m/s. Results also highlight that there exist optimal structural parameters for beam-film coupled structure to generate higher electrical output in addition to peak voltage to further enhance the performance of the proposed harvester compared to typical cantilever structure.

#### 4.2.3. Effect of impact parameters.

**Fig 11:**
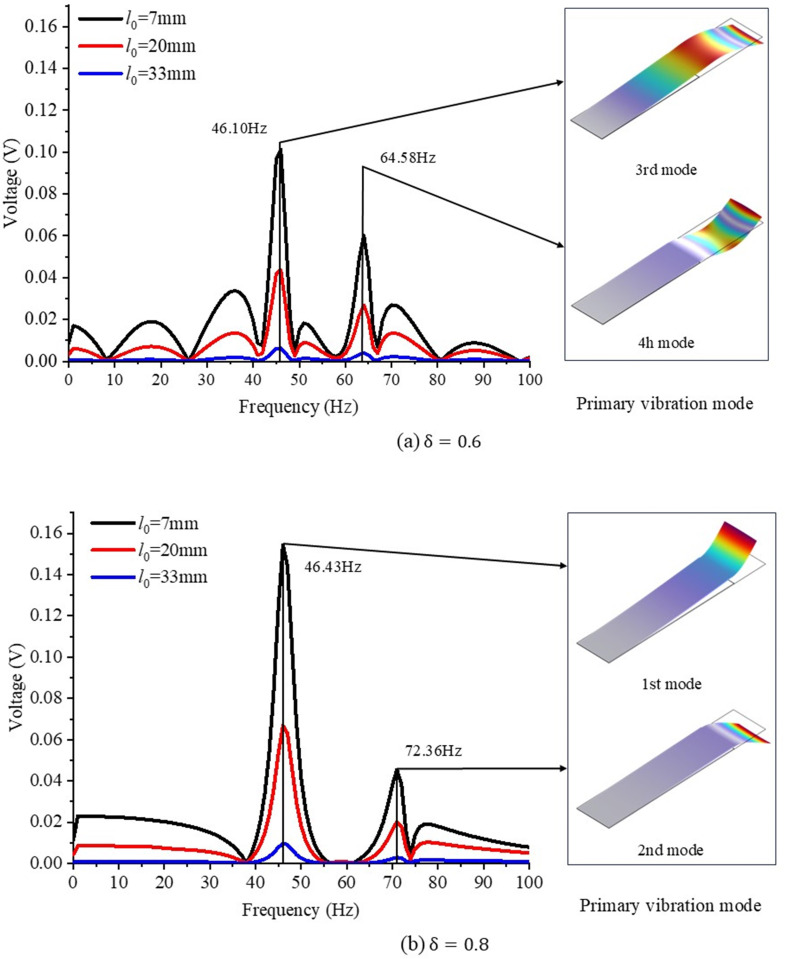
Comparison of voltage-frequency response curves and dominant vibration modes between film ratios 0.6 and 0.8 for different impact positions of water droplets.

Focus on film ratios of 0.6 and 0.8, the harvester is first tested with different impact positions $l_{0}$ (seen in Fig 4), and the frequency domain curve of output voltage is shown in Fig 11. Results show that as the impact position $l_{0}$ moves from near the beam end (7mm) to the fixed end (33mm), the peak voltage generated decreases significantly, especially for the film ratio *δ* = 0.8. As shown in Fig 11a, the peak frequency for *δ* = 0.6 is 46.1Hz and 64.5Hz, corresponding to the 3^rd^ and 4^th^ vibration mode of the harvester respectively. By comparison, Fig 11b shows that the peak frequency for *δ* = 0.8 is 46.43Hz and 72.36Hz, corresponding to the 1^st^ and 2^nd^ vibration mode respectively. Specifically, the voltage of film ratio 0.8 is dominated by the lower order vibration (46.43Hz) of the harvester, while the multi-mode vibration characteristics of the harvester is more pronounced for *δ* = 0.6. Although film ratios 0.6 and 0.8 result in different vibration modes of the harvester, the film-coupled structure deforms in a similar way for any impact position of water droplet. At a point view of energy harvesting, harvester of ratio 0.8 with an impact position close to the beam end (*l*_0_ = 7mm) can generate higher peak voltage, which further enhances better performance on energy density for a film ratio of 0.8 as shown in Fig 10.

**Fig 12:**
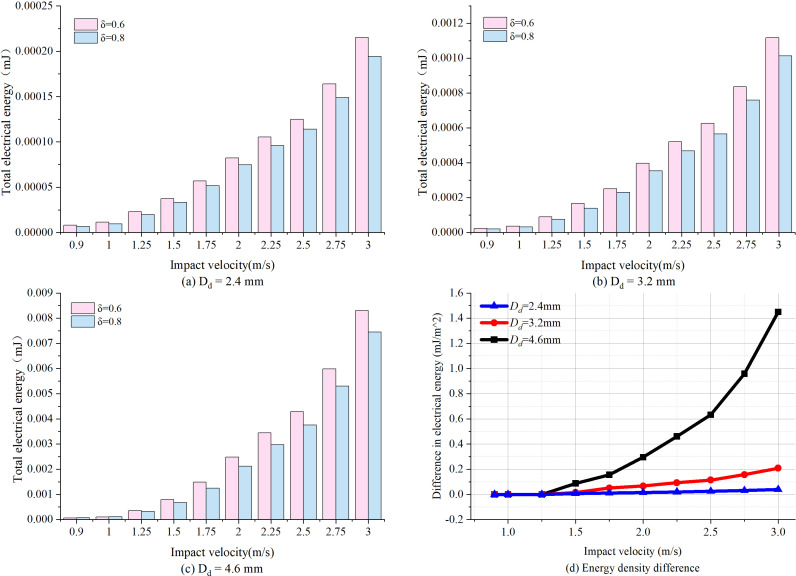
Comparison of total energy collected between film ratio of 0.6and 0.8 for (a) Droplet diameter 2.4mm; (b) Droplet diameter 3.2mm; (c) Droplet diameter 4.6mm and (d) The difference in energy collected between 0.6 and 0.8 for different impact parameters.

Apart from impact position, for other impact parameters (i.e., droplet diameter and impact velocity), the total energy collected after single droplet impact for film ratios 0.6 and 0.8 are calculated and compared in Fig 12. As shown in Fig 12a–12c, the total electrical energy of *δ* = 0.6 is generally higher than that of *δ* = 0.8 for any impact parameters, showing inconsistent results from Figs 10 and 11. It is related to the fact that smaller film ratio of 0.6 leads to a lower structural stiffness of the harvester as well as a lower vibration frequency of the harvester. In this case, although the peak value of electrical output is relatively decreased for film ratio of 0.6 (seen in Fig 11), the total energy collected is higher compared to the film ratio of 0.8, and the difference between them progressively increases with the impact velocity.

Fig 12d illustrates the variation of difference in energy density between film ratios 0.6 and 0.8 with the impact velocity for different droplet diameters 2.4mm, 3.2mm and 4.6mm. It can be seen that for smaller droplet diameters (e.g., 2.4mm and 3.2mm), the difference is insignificant. By comparison, for larger droplet diameters (e.g., $D_{\text{d}}$ = 4.6mm), the difference between 0.6 and 0.8 remains small when $V_{d}$ < 1.25m/s, however, the difference increases rapidly when $V_{d}$> 1.25m/s, reaching a maximum of 1.45 × 10^-6^mJ/m^2^ at a velocity of $V_{d}$ = 3.0m/s. Overall, for small impact velocity ($V_{d}$ < 1.25m/s), the difference of energy collected between 0.6 and 0.8 is insignificant. For larger droplet with important impact velocity, harvester with film ratio of 0.6 has significant advantages in generating higher energy collected compared to harvester with film ratio of 0.8. This also enhances the superiority of harvester with film ratio of 0.6 under heavy rainfall conditions toward rain energy harvesting utilizations.

## 5. Conclusion

Based on cantilever-style vibration energy harvesters, a PVDF beam-film coupled structure is designed and investigated for droplet-based harvesters. A Gaussian-type droplet force model is established by considering both the effect of structural and impact parameters. By applying the force model, a simulation model is constructed in COMSOL and validated by comparing with impact experiments under various impact parameters. The dynamic response and electromechanical output of the proposed harvester are investigated by use of the simulation model where the coupling effect of structural and impact parameters is mainly discussed. Main conclusions are summarized as follows:

1. Following the impulse theorem, the impact force of droplet is determined by using a Gaussian function. It relies on the equivalent stiffness of the coupled harvester and can accurately represent the variation of the impact force over time. By comparison with experimental results, the linearity between the open-circuit voltage and the displacement of the beam end is confirmed hence the simulation model coupled with the force model is also validated.
2. The beam-film coupled configuration of the harvester is mainly characterized by the film ratio (*δ*), which has a profound impact on electrical output and vibration modes of the harvester. As the film ratio increases, the harvester transits from single-mode to multi-mode vibration with a gradual reduction in response frequency. Lower film ratios (e.g., 0.5 and 0.6) are particularly suitable for harvesting ultra-low-frequency vibrations. The impact position ($l_{0}$) of droplet has little effect on the vibration modes of the harvester for all film ratios.
3. Although the peak voltage decreases as the film ratio increases, film ratios 0.6 and 0.8 exhibit higher energy density than other film ratios which provide an insight into further optimization design. For higher impact velocity 3.0m/s, film ratio 0.8 performs much better than film ratio 0.6 in energy density, showing a relatively great potential in device miniaturization and piezoelectric material efficiency improvement. However, the film ratio 0.6 always generates greater total energy collected than that of film ratio 0.8, and the difference between them becomes more significant for larger droplet size (e.g., $D_{d}$ > 3.2mm) and higher impact velocity ($V_{d}$> 1.25m/s). These results show that the proposed harvester with a ratio of 0.6 is more favorable in heavy rainfall conditions toward rain energy harvesting utilizations.
